# The Peri-Implant Microbiome—A Possible Factor Determining the Success of Surgical Peri-Implantitis Treatment?

**DOI:** 10.3390/dj12010020

**Published:** 2024-01-22

**Authors:** Jarno Hakkers, Lei Liu, Diederik F. M. Hentenaar, Gerry M. Raghoebar, Arjan Vissink, Henny J. A. Meijer, Lisa Walters, Hermie J. M. Harmsen, Yvonne C. M. de Waal

**Affiliations:** 1 Department of Oral and Maxillofacial Surgery, University Medical Center Groningen, University of Groningen, P.O. Box 30.001, 9700 RB Groningen, The Netherlands; d.f.m.hentenaar@umcg.nl (D.F.M.H.); g.m.raghoebar@umcg.nl (G.M.R.); a.vissink@umcg.nl (A.V.); h.j.a.meijer@umcg.nl (H.J.A.M.); 2 Department of Medical Microbiology and Infection Prevention, University Medical Center Groningen, University of Groningen, P.O. Box 30.001, 9700 RB Groningen, The Netherlands; l.liu@umcg.nl (L.L.); l.walters@umcg.nl (L.W.); h.j.m.harmsen@umcg.nl (H.J.M.H.); 3 Center for Dentistry and Oral Hygiene, University Medical Center Groningen, University of Groningen, P.O. Box 30.001, 9700 RB Groningen, The Netherlands; y.c.m.de.waal@umcg.nl

**Keywords:** peri-implantitis, microbiology, dental implant, surgical peri-implantitis treatment

## Abstract

The objective was to assess the effect of peri-implantitis surgery on the peri-implant microbiome with a follow-up of one year. A total of 25 peri-implantitis patients in whom non-surgical treatment has failed to solve peri-implantitis underwent resective surgical treatment. Their peri-implant pockets were sampled prior to surgical treatment (T0) and one year post treatment (T12). The natural dentition was sampled to analyse similarities and differences with the peri-implantitis samples. Treatment success was recorded. The change in microbial relative abundance levels was evaluated. The microbiota was analysed by sequencing the amplified V3-V4 region of the 16S rRNA genes. Sequence data were binned to amplicon sequence variants that were assigned to bacterial genera. Group differences were analysed using principal coordinate analysis, Wilcoxon signed rank tests, and *t*-tests. Beta diversity analyses reported a significant separation between peri-implantitis and natural dentition samples on T0 and T12, along with significant separations between successfully and non-successfully treated patients. *Eubacterium* was significantly lower on T12 compared to T0 for the peri-implantitis samples. *Treponema* and *Eubacterium* abundance levels were significantly lower in patients with treatment success on T0 and T12 versus no treatment success. Therefore, lower baseline levels of *Treponema* and *Eubacterium* seem to be associated with treatment success of peri-implantitis surgery. This study might aid clinicians in determining which peri-implantitis cases might be suitable for treatment and give a prognosis with regard to treatment success.

## 1. Introduction

Peri-implantitis is an inflammatory process resulting in the loss of bone surrounding dental implants. Pathogenic infection and a gradual decrease in commensal flora seem to predominate when an implant shifts from a state of health to a state of disease [1,2,3,4,5].

The presence of a biofilm and factors that facilitate plaque retention around an implant seem to be major contributors to the initiation and maintenance of peri-implant pathology [6]. A number of studies on the microbiological composition of such biofilms have been undertaken, and several methodologies to analyse the microbiota in peri-implant biofilms are described [4,5,7,8]. Close-ended methods like polymerase chain reaction (PCR), which uses specific primers, require prior knowledge of the microbiome associated with the sample. Methods like the checkerboard DNA–DNA hybridization technique developed by Socransky (1994) use a predetermined whole genome probe to hybridize the pathogen’s DNA against the probe [9]. Nowadays, open-ended methods are used, such as amplicon sequencing of the 16S rRNA gene.

The advancement of 16S rRNA gene sequencing has enabled comparative studies of peri-implant health versus disease and the progression of peri-implant pathology. Considerable microbial differences were observed between diseased and healthy implants. Species such as *Porphyromonas gingivalis*, *Tannerella forsythia*, *Treponema denticola*, *Fusobacterium nucleatum*, and *Prevotella intermedia* seem to be associated with peri-implantitis [10]. Peri-implantitis also seems to comprise a lower diversity of microbiota compared to periodontal sites and healthy implants [5,11]. Conventional bacterial analysis techniques show similarities in periodontopathic microorganisms present in both peri-implantitis and periodontitis cases [12]. However, the core microbiota between the two inflammatory processes seems to differ in that peri-implantitis seems to consist of Gram-negative anaerobic periopathogens and opportunistic microorganisms and is frequently associated with nonsaccharolytic anaerobic Gram-positive rods [13,14,15]. Current Next-Generation Sequencing (NGS) techniques have established that the distinct microbial community in peri-implantitis consists of both periodontal pathogens and site-specific pathogens [5,16].

The aim of peri-implantitis treatment is to stop the inflammatory process and re-establish a healthy peri-implant environment [17]. Microbial analysis following surgical treatment modalities could give an insight into the change in the composition of the peri-implant microbiome over time. The available literature is inconclusive on the effect of surgical peri-implantitis treatment on the peri-implant microbiome and its effects on disease advancement. Studies have shown promising short-term results of surgical peri-implantitis treatment on the peri-implant microbiome. Red complex species, along with *Fusobacterium nucleatum* subsp. *Nucleatum*, e.g., were reduced 3 months after performing open flap debridement of the implant surface [18]. Unfortunately, this reduction may be temporary in nature since the recurrence of pathogens after 6 to 12 months has also been observed [19]. Therefore, a proper evaluation of the effects of peri-implantitis surgery on the peri-implant microbiome is needed. The aim of this study was to investigate the effect of peri-implantitis surgery on the submucosal peri-implant microbiota using 16S rRNA gene sequencing techniques with a follow-up of 1 year. The secondary aim was to assess differences between patients with a successful or non-successful clinical surgical peri-implantitis treatment outcome.

## 2. Materials and Methods

### 2.1. Study Design and Setting

In a convenience sample of 25 consecutive dentate patients without periodontitis (absence of probing depths > 5 mm with concomitant bleeding on probing (BoP), overall plaque and bleeding scores ≤ 20%) harbouring a total of 25 implants diagnosed with peri-implantitis (peri-implant probing depth (PD) ≥ 5 mm with concomitant BoP and/or suppuration on probing (SoP), progressive loss of marginal bone (MBL) ≥ 2 mm, when compared to the baseline radiograph), the effect of surgical treatment was studied after non-surgical treatment has failed to resolve the peri-implantitis. Exclusion criteria were as follows: history of local head and neck therapy, pregnancy and/or lactation, uncontrolled diabetes mellitus (HbA1c > 7% or >53 mmol/mol), use of antibiotics within 2 months before baseline assessment, known allergy to chlorhexidine, long-term use of inflammatory drugs, incapability of performing basal oral hygiene measures, implants with bone loss exceeding 2/3 of the implant length, implant mobility, chronic bronchitis, and/or asthma. Microbiological samples were collected from December 2016 until February 2020 by one researcher (DH). All treatments were performed at the department of Oral and Maxillofacial Surgery of the University Medical Center Groningen, The Netherlands (UMCG). The study design was approved by the ethical committee of the UMCG with study number 2016/356, registered in the Dutch national trial register under the number NL8621, and was conducted in accordance with the Declaration of Helsinki. All patients were asked to sign an informed consent form prior to enrolling in the study.

### 2.2. Outcomes

The changes in microbial relative abundance levels (%) of the peri-implant microbiota between T0 (prior to surgical peri-implantitis treatment) and T12 (one year after surgical peri-implantitis treatment) were analysed. The periodontium surrounding the natural dentition was also sampled to evaluate similarities or differences in the presence of the bacterial genera observed in the peri-implantitis samples. A sub-analysis regarding the differences in microbial relative abundance levels (%) of the peri-implant microbiota between patients who achieved treatment success and patients who did not achieve treatment success was also performed. Treatment success was defined as follows:

- Absence of PD ≥ 5 mm with concomitant BoP;
- Absence of SoP;
- No progressive MBL ≥ 0.5 mm on T12 compared to the baseline radiograph taken on T0.

### 2.3. Clinical and Radiographic Parameters

Clinical parameters such as peri-implant and periodontal PD, midbuccal recession (REC), bleeding score (BS), and suppuration score (SS) were scored at six sites per implant or tooth using a Hu-Friedy PCPUNC156 periodontal probe (HuFriedyGroup, Chicago, IL, USA). Peri-implant marginal bone levels were measured mesially and distally on calibrated radiographs using DICOM software (DicomWorks 1.5). Bone loss was assessed by comparing bone levels at T0 and T12.

### 2.4. Surgical Procedure

The surgical peri-implantitis treatment was performed using local anaesthetics. After performing an incision ≥1 mm apical of the mucosal margin, in order to better facilitate the removal of the inflamed soft tissue collar and create pocket reduction, a full-thickness flap was raised buccally and lingually, and the implant surface was exposed. Granulation tissue was removed using titanium curettes (Hu Friedy^®^, Chicago, IL, USA). Calculus, if present, was carefully removed with a scaler tip. The implant surface was cleaned using an air-polishing device with erythritol-based powder and hand instruments combined with gauzes soaked in saline solution until the implant surface was deemed to be clean by the surgeon. After repositioning the mucosal flap with single interrupted sutures, the patient was given postoperative instructions. Patients were instructed to use an antiseptic mouthwash (0.2% chlorhexidine, Orasol^®^, ICM Pharma Pte. Ltd., Singapore) for 2 weeks after surgery, two times daily. Two weeks after surgery, the sutures were removed, and further oral hygiene instructions were given (twice daily use of an electric toothbrush and use of interdental brushes). The patients were recalled 3, 6, 9, and 12 months after the treatment for a re-examination and peri-implant maintenance therapy.

### 2.5. Microbial Sample Collection

The peri-implantitis (IMPL) samples were collected from four sites per implant (mesiobuccal, distobuccal, mesiolingual, and distolingual) using paper points (Antaeos #25, Charles B Schwed Co., LLC, Danbury, CT, USA). After removing the supragingival plaque and air drying the sampling site, the paper point was inserted into the pocket and left there for 15 s. Four paper points per implant were pooled as one sample in a coded 1.5 mL microtube containing 1 mL of reduced transport fluid (RTF). Sampling of the natural dentition (ND) was performed by administering a paper point into the deepest periodontal pocket in each quadrant. If no deep pockets could be identified, the mesiobuccal pocket of the first molar was sampled. The four paper points were pooled in one screw-cap cup. The samples were stored at −80 °C. This procedure was performed at baseline (three months after non-surgical treatment, just before surgical treatment, T0) and 12 months after surgical treatment (T12).

### 2.6. DNA Isolation and Sequencing

In preparation for DNA isolation, the samples were vortexed for 20 s. Two paper points per sample and 500 µL of RTF were transferred to a 2 mL screw cap tube containing 0.5 g of 0.1 mm zirconia beads and four 3 mm glass beads. Oscillation was performed three times for 1 min at 5.5 m/s, with 30 s intervals, in a Precellys 24 (Bertin Instruments, Montigny-le-Bretonneux, France). The samples were incubated for 15 min at 95 °C and then centrifuged for 5 min at 14,000 rpm and 4 °C. The supernatant was transferred to a new 1.5 mL cup.

The QIAamp DNA mini kit (Qiagen, Hilden, Germany, #51306) was used for DNA extraction in accordance with the manufacturer’s protocol for DNA purification from buccal cotton swabs, following the protocol for cotton swabs. The following adjustments were made to the protocol: after the protease K incubation step, 4 µg/mL RNase was added and incubated for 30 min at 37 °C. To increase the yield, the volume of buffer AE was decreased to 100 µL, and an extra elution step was added. The DNA concentration was measured using Nanodrop 2000 (Thermo Fischer Scientific, Waltham, MA, USA).

The V3-V4 regions of the 16S rRNA gene were amplified by PCR using modified 341F and 806R primers (Eurogentec, Seraing, Belgium) containing adapter sequences (Miseq, Illumina) and a six-base pair barcode (see Supplementary Materials). The following mastermix was used, with a final volume of 50 µL: 1X Phire Hot Start II Master Mix (Thermo Fisher Scientific, Waltham, MA, USA), 0.2 µM 341F primer, 0.2 µM 806R primer, 5 µL DNA, and nuclease-free water. The PCR programme was as follows: 98 °C for 30 s, a total of 33 cycles at 98 °C for 5 s, 50 °C for 5 s, 72 °C for 10 s, followed by 72 °C for 1 min, and ended at 4 °C until further use. Amplicons were detected via agarose gel electrophoresis.

The DNA library was purified using AMPure XP beads (Beckman Coulter, Brea, CA, USA) according to the manufacturer’s protocol with a few adjustments: 80% ethanol was used, the beads were air dried for 15 min after the second wash step, and 10 mM Tris HCl buffer, pH 8.5, was used for elution. DNA concentrations were measured with the Qubit dsDNA HS assay kit and Qubit 2.0 fluorometer (Invitrogen, Waltham, MA, USA). The libraries were normalised to 2 nM using Tris-EDTA buffer pH 8.0, and 5 µL of each was pooled together. The library was denatured and diluted, and PhiX control was added according to the manufacturer’s protocol (Miseq, Illumina, San Diego, CA, USA). Sequencing was performed using the Miseq Reagent Kit V3, 600 cycles (Outllumina, San Diego, CA, USA, #MS-102-3003), and a Miseq benchtop sequencer (Illumina).

The paired-end reads, demultiplexed based on the barcode, were retrieved from the Illumina platform and instructed by the EasyAmplicon analysis pipeline [20]. The joined reads were quality-controlled with a maximum error rate of 1%, and the primer sequences were cut by VSEARCH [21]. Denoising (removing chimeric sequences, removing singletons, and dereplication) was done with USEARCH and VSEARCH [22]. The amplicon sequence variants (ASVs) were assigned based on Ribosomal Database Project Set 16 with an RDP classifier [23]. The raw sequencing data have been submitted to the National Center for Biotechnology Information (NCBI) Sequence Read Archive (SRA) under accession number BioProject PRJNA1050775.

### 2.7. Statistical Analysis

The multivariate analysis of the 16S rRNA gene sequencing data was performed after normalization using cumulative sum scaling. The alpha (Chao, Shannon, and ASV richness index) and beta diversity calculations were executed using QIIME [24]. The Bray–Curtis distance of the beta diversity was calculated and represented in a principal coordinate analysis (PCoA) and performed using the R package “stats”. The ADONIS function in the “vegan” package tested significantly between groups with 999 permutations.

Changes in microbial relative abundance levels in the IMPL samples and the ND samples between T0 and T12 were analysed using Wilcoxon’s signed-rank test. The T0 and T12 differences between the IMPL and ND samples were analysed using the Student’s *t*-test. Normality testing was performed with Kolmogorov–Smirnov tests. During the analysis, smoking was analysed as a possible confounder, as it could affect the composition of the oral microbiome [25]. The mean values for the clinical parameters were analysed using independent sample *t*-tests.

Descriptive statistics were performed using IBM SPSS Statistics 28 (International Business Machines Corporation, Armonk, NY, USA). The software R (version 4.0.2) for Windows 10 was used to perform the statistical analyses and data visualization of the microbiological samples. *p*-values < 0.05 were considered to be statistically significant and were adjusted for multiple testing using the Benjamini-Hochberg method.

## 3. Results

### 3.1. Sample Processing Results

**Figure 1:**
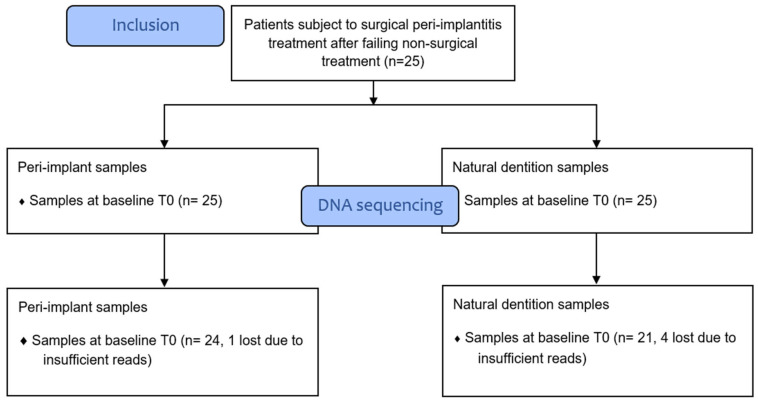
Flow chart on sample processing and DNA sequencing. The colours are only used to differentiate between the timepoints and the sample origins, being either ND or IMPL.

**Figure 2:**
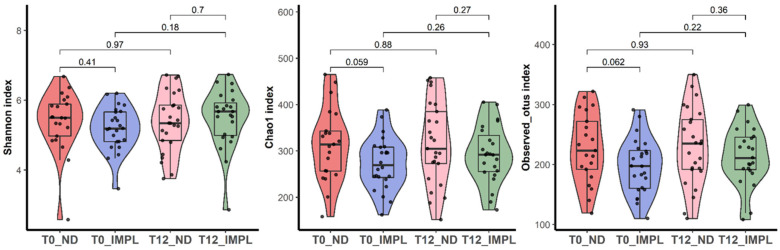
Comparison of Shannon index, Chao1 index, and the ASV richness for the ND and IMPL samples per time point.

**Figure 3:**
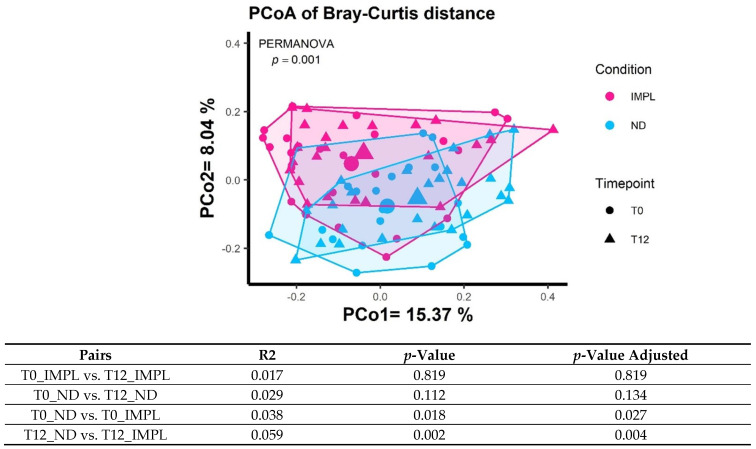
PCoA of Bray–Curtis distance with the implant samples (IMPL) and the natural dentition samples (ND) on T0 and T12.

**Figure 4:**
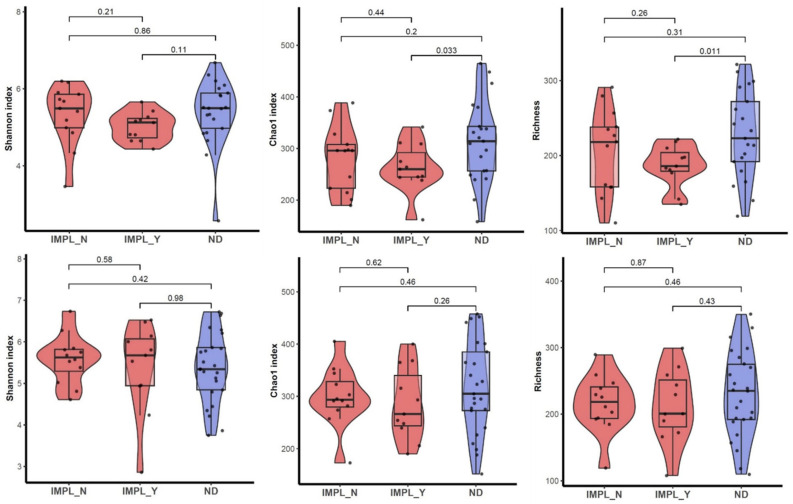
Alpha diversity (Shannon, Chao1, and ASV richness) on the peri-implantitis treatment success on T0 (**top**) and T12 (**bottom**). IMPL_N = no treatment success, IMPL_Y = treatment success, and ND = natural dentition.

**Figure 5:**
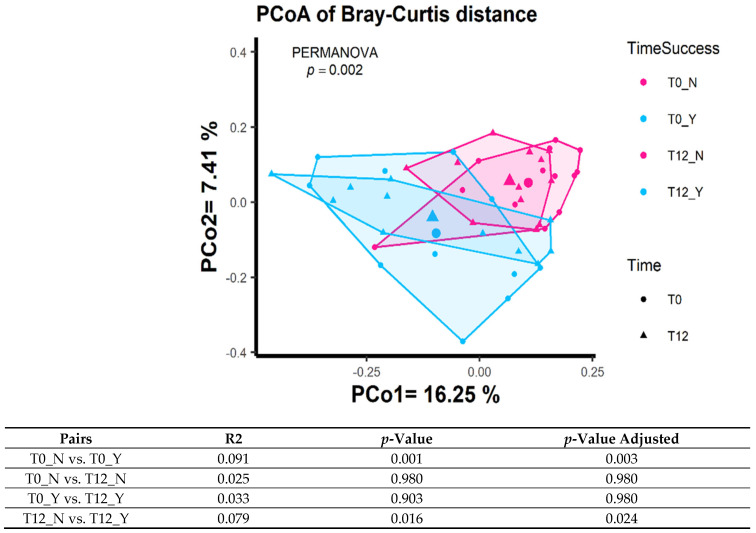
PCoA of Bray–Curtis distance with regard to treatment success (Y) and no treatment success (N) in the IMPL samples.

Sample processing and quality control results are presented in Figure 1. The patient characteristics are described in Table 1. A total of 1,697,851 reads were generated after processing. No significant differences were observed with regard to the alpha diversity (Chao1 index, Shannon index, and ASV richness, Figure 2), indicating that sample diversity, sample richness, and the observed operational taxonomic units (OTU) overall did not significantly differ between the IMPL and ND samples. After analysis, smoking could not be classified as a confounder in this study population. The overall principal components analysis (Figure 3) showed a significant separation between the IMPL and ND samples at T0 and T12. More specifically, there was a significant separation between the IMPL and the ND samples on T0 and the IMPL and the ND samples on T12. No significant separation could be observed between the T0 and T12 IMPL samples and the T0 and T12 ND samples. Alpha diversity analyses separated by treatment success (yes/no) on T0 and T12 for the IMPL samples showed a significant difference between the successfully treated IMPL samples, i.e., absence of PD ≥ 5 mm with concomitant BoP, absence of SoP, and no progressive MBL ≥ 0.5 mm on T12 compared to the baseline radiograph taken on T0, and the ND samples with regard to the Chao1 (*p* = 0.033) and ASV richness (*p* = 0.011) (Figure 4) on T0. Apart from these, the analyses did not show differences in alpha diversity. The principal component analysis (Figure 5) showed a significant separation between treatment success and no treatment success at both T0 and T12. In contrast, no significant separation could be observed in treatment success between T0 and T12, and in no treatment success between T0 and T12.

### 3.2. Abundance Levels

**Figure 6:**
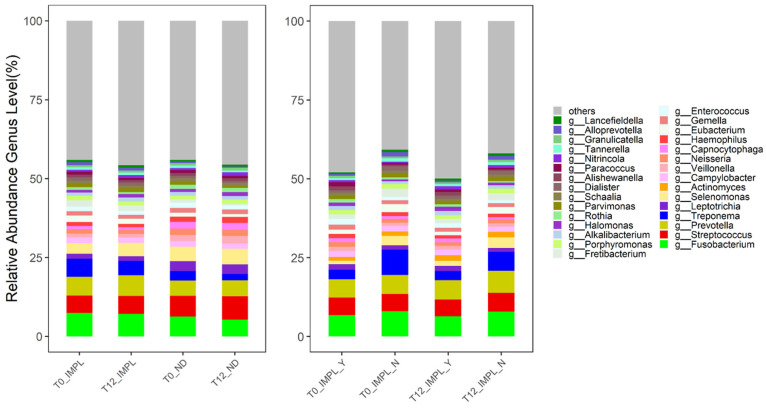
Mean relative abundance levels of natural dentition (ND) and implant (IMPL) samples on T0 and T12 per group (**left**) and subdivided into successfully (Y) and non-successfully (N) treated patients per group (**right**). The top 30 genera were coloured.

The charts in Figure 6(left) show the results with regard to the mean relative abundance levels of the IMPL and ND samples separated for T0 and T12. *Eubacterium* was significantly reduced on T12 in the IMPL samples (*p* = 0.046). The mean relative abundance levels among the patients with a successful or non-successful clinical treatment outcome are shown in Figure 6(right). At T12, the IMPL samples obtained from successfully treated patients contained significantly lower relative abundance levels of *Treponema-* (*p* = 0.002) and *Eubacterium*- (*p* = 0.004) species than the implants with a non-successful treatment outcome. To assess whether these genera could serve as predictors for clinical treatment success, the IMPL samples taken at T0 were added to the comparative analyses of treatment success. At T0, the mean relative abundance levels of *Treponema* (*p* = 0.003) and *Eubacterium* (*p* = 0.009) were also significantly lower in the successfully treated patients (together with *Selenomonas* (*p* = 0.001), *Fretibacterium* (*p* = 0.039), and *Dialister* (*p* = 0.035)), which means that the abundance levels of *Eubacterium* and *Treponema* were significantly lower at both T0 and T12 in successfully treated patients. Regarding the clinical parameters, mean peri-implant PD was significantly lower in the successfully treated patients compared to the non-successfully treated patients on both T0 (4.1 mm vs. 5.5 mm, respectively, *p* < 0.001) and T12 (2.7 mm vs. 4.4 mm, respectively, *p* < 0.001).

## 4. Discussion

This study assessed the peri-implant microbiota prior to and 12 months after peri-implantitis surgery in patients in whom non-surgical treatment has failed to resolve the peri-implantitis. In addition, analyses regarding the differences between patients with a successful or non-successful clinical treatment outcome were performed. Overall, minor differences could be observed between the peri-implant microbiome on the baseline and on T12, indicating that one year after peri-implantitis surgery, the overall composition of the peri-implant microbiome seems to be predominantly similar to the baseline levels. However, when subdivided into treatment success, the peri-implant microbiome in patients who were successfully treated was slightly different from that of patients who were not successfully treated, indicating that specific genera could play a vital role in determining whether a patient can be successfully surgically treated for peri-implantitis or not.

Alpha diversity analyses reported no significant differences in sample richness, sample diversity, or OTU’s between the IMPL and the ND samples on T0 and T12 overall. These findings are in line with the study conducted by Barbagallo et al. (2021), who did not find statistically significant differences with regard to alpha diversity indices between healthy periodontal samples, periodontitis samples, and peri-implantitis samples [26]. Analysing the sample contents in detail shows that overall, only the *Eubacterium* species seemed to have reduced significantly in the peri-implantitis samples one year postoperatively. This bacterial genus has previously been associated with peri-implant pathology, as *Eubacterium nodatum*, *Eubacterium brachy*, and *Eubacterium saphenum* were highly abundant at peri-implantitis sites [27].

Zheng et al. (2015) reported higher relative abundance levels of *Eubacterium* species at peri-implantitis locations and found that the presence of *Eubacterium minutum* correlated with the presence of *Prevotella intermedia*, suggesting an association between *Eubacterium* and peri-implantitis [28].

Strikingly, the abundance levels of *Eubacterium* and *Treponema* species were significantly lower in patients who met the criteria for treatment success. The *Eubacterium* species belong to the group of asaccharolytic anaerobic Gram-positive rods (AAGPRs). These species are capable of producing butyrate, which can disturb host cell function and has, therefore, been associated with periodontitis and peri-implantitis [15,29,30,31]. *Treponema* spp., e.g., *Treponema denticola*, seem to be common at peri-implantitis sites [7,32]. Therefore, the reduction in spirochaetes, e.g., *Treponema* spp., might also contribute to the long-term submission of peri-implant pathology [33]. These genera were already significantly less abundant at T0, which could also underline the notion that the presence of certain genera might serve as prognostic indicators when predicting the chance of treatment success.

Chao1 and the ASV richness were significantly higher in the ND samples when compared to successfully treated IMPL samples at baseline but not compared to the non-successfully treated IMPL samples. Belibasakis and Manoil (2020) describe an increase in microbial diversity as the severity of peri-implant inflammation intensifies [5]. It could be hypothesized that in this study, the microbiota of implants that were treated successfully were at an earlier stage of peri-implantitis development at baseline, characterized by a higher relative abundance of early pathogenic colonizers. This could account for the lower diversity. When peri-implantitis is then allowed to progress without intervention, the peri-implant microbiota could develop and mature into a more complex ecosystem, which could increase its diversity. This could indicate that the baseline peri-implantitis microbiota associated with treatment success is characterised by a lower microbial diversity when compared to healthy periodontal samples due to a higher abundance of *Eubacterium* and *Treponema* species in samples that demonstrate lower community richness.

Furthermore, both *Treponema* and *Eubacterium* contain species that proliferate under anaerobic circumstances [34,35], such as deep peri-implant pockets. The mean peri-implant PD was significantly deeper in the non-successfully treated patients and could have enabled *Treponema* and *Eubacterium* species to multiply. It could, therefore, be questioned whether the presence of these genera has caused the peri-implantitis surgery to be less successful or if this is due to deeper baseline pocket depths. Regardless, *Treponema* and *Eubacterium* seem to have thrived in patients with deeper baseline peri-implant PD and might, therefore, be associated with persistent peri-implantitis.

Only a few studies have evaluated peri-implant microbiology in relation to a therapeutic intervention over time. Leonhardt et al. (2003) studied surgical peri-implantitis treatments with personalized adjunctive antibiotic regimens and evaluated the peri-implant microbiota over a period of 5 years using agar plate inoculations [36]. They observed a reduction in *Aggregatibacter actinomycetemcomitans* and enteric rods after 5 years. They also noticed that, although *Prevotella intermedia* had decreased after 1 year, there was an increase 5 years postoperatively. Whether an antibiotic regimen specifically targeted towards peri-implant *Treponema* or *Eubacterium* species could benefit treatment success has not yet been investigated. In the treatment of periodontitis, the bactericidal effects of metronidazole against anaerobes, including spirochaetes and Gram-negative and Gram-positive bacterial species, have proven to be effective [37]. With regard to *Treponema* specifically, Okamoto-Shibayama et al. (2017) assessed the in vitro susceptibility of several *Treponema* species to different antibiotics and found that doxycycline, minocycline, azithromycin, and erythromycin were effective against all *Treponema* species tested [38]. This could provide a basis to further examine the position of target-specific antimicrobials in the treatment of peri-implantitis. Whether it is at all possible to implement an antibiotic regimen remains to be explored, as the peri-implantitis ecosystem has proven to be a complex, mixed infection.

Isehed et al. (2016) executed a randomized clinical trial on the use of enamel matrix derivative in peri-implantitis surgery [19]. They took microbial samples at baseline, 2 weeks, 3, 6, and 12 months postoperatively. At baseline, high abundance levels of species similar to the bacterial genera found in this study were observed: *Fusobacteria* (cluster probe), *Parvimonas micra, Porphyromonas* sp. HOT279, *Eubacterium nodatum, Porphyromonas gingivalis, Tannerella forsythia*, and *Campylobacter rectus/Campylobacter concisus*. However, the majority of the species that were reduced at 2 weeks and 3 months had returned to levels similar to the baseline levels at 12 months. These findings are in line with the results in this study, indicating that the reduction in abundance levels might be temporary in nature since the majority of the observed pathogens in this cohort did not differ significantly in abundance levels between the baseline and T12 peri-implantitis samples. However, whether the presence of these bacteria induces an inflammatory reaction could also be dependent on host responses. Wang et al. (2021) identified peri-implantitis risk groups with specific immune profiles, stating that the peri-implant immune microenvironment shapes the peri-implant microbial composition [39]. Accordingly, it is worth including this insight in future research focusing on the role of the peri-implant microbiome with respect to peri-implantitis treatment.

This study has several limitations. For one, no microbiological samples were taken from the implants diagnosed with peri-implantitis prior to non-surgical treatment. Therefore, the effect of the non-surgical treatment on the peri-implant microbiome could not be assessed. The starting point of this study was the microbiome of patients in whom non-surgical treatment has failed to resolve the peri-implantitis just before commencing surgical treatment. Furthermore, the criteria that define treatment success are based on the consensus report by the Seventh European Workshop on Periodontology [40]. This is due to the fact that the aforementioned clinical trial was conducted prior to the publication of a new classification on peri-implant pathology in 2017 [41]. It is worth noting that these criteria are comparable to the recommended end points of successful surgical therapy for peri-implantitis, as stated in the EFP S3 level clinical practice guideline [17]. As the present study encompassed the evaluation of a treatment intervention, no healthy implant controls could be included because the paired-sample inclination of the methodology did not always allow for a healthy implant to be present in the same patient. Lastly, this study included patients with different types of prosthetic rehabilitations, including single crowns, fixed partial dentures, and overdentures, which might have had an impact on the clinical and microbiological outcomes. Single crowns predominated in this cohort, comprising 22 of the 25 included patients.

## 5. Conclusions

In summary, this study brought the genera *Treponema* and *Eubacterium* into focus with regard to their importance when trying to achieve treatment success after performing surgical peri-implantitis therapy. Deeper baseline peri-implant pocket depths might facilitate the growth of species belonging to these genera, which could negatively influence surgical peri-implantitis treatment success. Future prospective research could further elaborate on the role of *Treponema* and *Eubacterium* and whether the reduction in abundance levels of these genera could significantly aid in achieving disease resolution. Clinicians may benefit from having advance knowledge of the peri-implant microbiome for the purpose of determining the most appropriate implant treatment.

## Figures and Tables

**Table 1:** Patient characteristics.

Characteristics	Overall Patient Outcome (*n* = 25)	Successfully Treated Patients (*n* = 12)	Non-Successfully Treated Patients (*n* = 13)
Gender (m/f)	16/9	7/5	9/4
Mean age (SD)	56.3 (12.32)	57.3 (9.3)	55.4 (14.9)
Smoking (y/n)	7/18	2/10	5/8
Suppurating implants on T0 (y/n)	12/13	5/7	7/6
Suppurating implants on T12 (y/n)	9/25	0/12	8/5
Mean peri-implant PD in mm on T0, six sites (SD)	4.8 (1.2)	4.1 (0.7)	5.5 (1.1)
Mean peri-implant PD in mm on T12, six sites (SD)	3.6 (1.1)	2.7 (0.5)	4.4 (1.0)
Mean peri-implant BoP on T0, % (SD)	61.3 (34.6)	56.9 (36.6)	65.4 (33.7)
Mean peri-implant BoP on T12, % (SD)	43.9 (28.0)	30.5 (22.3)	56.3 (27.7)
Mean peri-implant REC increase in mm between T0 and T12 (SD)	0.3 (1.1)	0.3 (1.4)	0.2 (0.7)
Mean periodontal PD in mm on T0, six sites (SD)	2.1 (0.3)	2.0 (0.3)	2.2 (0.3)
Mean periodontal PD in mm on T12, six sites (SD)	2.1 (0.2)	2.1 (0.3)	2.1 (0.2)
Mean periodontal BoP on T0, % (SD)	9.1 (7.1)	7.1 (3.5)	11.1 (9.0)
Mean periodontal BoP on T12, % (SD)	11.2 (7.9)	11.0 (7.8)	11.3 (8.2)

## Data Availability

The data presented in this study are available on reasonable request from the corresponding author.

## Supplementary Materials

The following are available online at https://www.mdpi.com/article/10.3390/dj12010020/s1.
