# Supplementary material for: The Peri-Implant Microbiome—A Possible Factor Determining the Success of Surgical Peri-Implantitis Treatment?
Source: Dent J (Basel). 2024 Jan 22;12(1):20. doi: 10.3390/dj12010020 (PMC10814184; doi:10.3390/dj12010020)
Supplement: Supplementary file 1 [file dentistry-12-00020-s001.zip › dentistry-2754795-supplementary.pdf]

V3F_modified		aatgatacggcgaccaccgagatct		acactctttccctacacgacgctcttccgatct	NNNNCCTACGGGAGGCAGCAG
V4R	1	caagcagaagacggcatacgagat	**ATCACG**	gtgactggagttcagacgtgtgctcttccgatct	GGACTACHVGGGTWTCTAAT
V4R	2	caagcagaagacggcatacgagat	**CGATGT**	gtgactggagttcagacgtgtgctcttccgatct	GGACTACHVGGGTWTCTAAT
V4R	3	caagcagaagacggcatacgagat	**TTAGGC**	gtgactggagttcagacgtgtgctcttccgatct	GGACTACHVGGGTWTCTAAT
V4R	4	caagcagaagacggcatacgagat	**TGACCA**	gtgactggagttcagacgtgtgctcttccgatct	GGACTACHVGGGTWTCTAAT
V4R	5	caagcagaagacggcatacgagat	**ACAGTG**	gtgactggagttcagacgtgtgctcttccgatct	GGACTACHVGGGTWTCTAAT
V4R	6	caagcagaagacggcatacgagat	**GCCAAT**	gtgactggagttcagacgtgtgctcttccgatct	GGACTACHVGGGTWTCTAAT
V4R	7	caagcagaagacggcatacgagat	**CAGATC**	gtgactggagttcagacgtgtgctcttccgatct	GGACTACHVGGGTWTCTAAT
V4R	8	caagcagaagacggcatacgagat	**ACTTGA**	gtgactggagttcagacgtgtgctcttccgatct	GGACTACHVGGGTWTCTAAT
V4R	9	caagcagaagacggcatacgagat	**GATCAG**	gtgactggagttcagacgtgtgctcttccgatct	GGACTACHVGGGTWTCTAAT
V4R	10	caagcagaagacggcatacgagat	**TAGCTT**	gtgactggagttcagacgtgtgctcttccgatct	GGACTACHVGGGTWTCTAAT
V4R	11	caagcagaagacggcatacgagat	**GGCTAC**	gtgactggagttcagacgtgtgctcttccgatct	GGACTACHVGGGTWTCTAAT
V4R	12	caagcagaagacggcatacgagat	**CTTGTA**	gtgactggagttcagacgtgtgctcttccgatct	GGACTACHVGGGTWTCTAAT
V4R	13	caagcagaagacggcatacgagat	**AGTACG**	gtgactggagttcagacgtgtgctcttccgatct	GGACTACHVGGGTWTCTAAT
V4R	14	caagcagaagacggcatacgagat	**TCAGTC**	gtgactggagttcagacgtgtgctcttccgatct	GGACTACHVGGGTWTCTAAT
V4R	15	caagcagaagacggcatacgagat	**TTGAGC**	gtgactggagttcagacgtgtgctcttccgatct	GGACTACHVGGGTWTCTAAT
V4R	16	caagcagaagacggcatacgagat	**AAGCGA**	gtgactggagttcagacgtgtgctcttccgatct	GGACTACHVGGGTWTCTAAT
V4R	18	caagcagaagacggcatacgagat	**GGTTGT**	gtgactggagttcagacgtgtgctcttccgatct	GGACTACHVGGGTWTCTAAT
V4R	19	caagcagaagacggcatacgagat	**TGAGGT**	gtgactggagttcagacgtgtgctcttccgatct	GGACTACHVGGGTWTCTAAT
V4R	20	caagcagaagacggcatacgagat	**TACCGT**	gtgactggagttcagacgtgtgctcttccgatct	GGACTACHVGGGTWTCTAAT
V4R	21	caagcagaagacggcatacgagat	**CCAACT**	gtgactggagttcagacgtgtgctcttccgatct	GGACTACHVGGGTWTCTAAT
V4R	22	caagcagaagacggcatacgagat	**AGAGAG**	gtgactggagttcagacgtgtgctcttccgatct	GGACTACHVGGGTWTCTAAT
V4R	23	caagcagaagacggcatacgagat	**CACTTG**	gtgactggagttcagacgtgtgctcttccgatct	GGACTACHVGGGTWTCTAAT
V4R	24	caagcagaagacggcatacgagat	**TCAAGG**	gtgactggagttcagacgtgtgctcttccgatct	GGACTACHVGGGTWTCTAAT
V4R	25	caagcagaagacggcatacgagat	**AGTGGT**	gtgactggagttcagacgtgtgctcttccgatct	GGACTACHVGGGTWTCTAAT
V4R	26	caagcagaagacggcatacgagat	**GACACT**	gtgactggagttcagacgtgtgctcttccgatct	GGACTACHVGGGTWTCTAAT
V4R	27	caagcagaagacggcatacgagat	**CCTTCT**	gtgactggagttcagacgtgtgctcttccgatct	GGACTACHVGGGTWTCTAAT
V4R	28	caagcagaagacggcatacgagat	**GGATAA**	gtgactggagttcagacgtgtgctcttccgatct	GGACTACHVGGGTWTCTAAT

V4R	29	caagcagaagacggcatacgagat	**CCTTAA**	gtgactggagttcagacgtgtgctcttccgatct	GGACTACHVGGGTWTCTAAT
V4R	30	caagcagaagacggcatacgagat	**CAAGAA**	gtgactggagttcagacgtgtgctcttccgatct	GGACTACHVGGGTWTCTAAT
V4R	31	caagcagaagacggcatacgagat	**GTTGAA**	gtgactggagttcagacgtgtgctcttccgatct	GGACTACHVGGGTWTCTAAT
V4R	32	caagcagaagacggcatacgagat	**TCACAA**	gtgactggagttcagacgtgtgctcttccgatct	GGACTACHVGGGTWTCTAAT
V4R	33	caagcagaagacggcatacgagat	**AGTCAA**	gtgactggagttcagacgtgtgctcttccgatct	GGACTACHVGGGTWTCTAAT
V4R	34	caagcagaagacggcatacgagat	**CGAATA**	gtgactggagttcagacgtgtgctcttccgatct	GGACTACHVGGGTWTCTAAT
V4R	35	caagcagaagacggcatacgagat	**GCTATA**	gtgactggagttcagacgtgtgctcttccgatct	GGACTACHVGGGTWTCTAAT
V4R	36	caagcagaagacggcatacgagat	**GAGTTA**	gtgactggagttcagacgtgtgctcttccgatct	GGACTACHVGGGTWTCTAAT
V4R	37	caagcagaagacggcatacgagat	**TTGGTA**	gtgactggagttcagacgtgtgctcttccgatct	GGACTACHVGGGTWTCTAAT
V4R	38	caagcagaagacggcatacgagat	**AACGTA**	gtgactggagttcagacgtgtgctcttccgatct	GGACTACHVGGGTWTCTAAT
V4R	39	caagcagaagacggcatacgagat	**GTACTA**	gtgactggagttcagacgtgtgctcttccgatct	GGACTACHVGGGTWTCTAAT
V4R	40	caagcagaagacggcatacgagat	**CATCTA**	gtgactggagttcagacgtgtgctcttccgatct	GGACTACHVGGGTWTCTAAT
V4R	41	caagcagaagacggcatacgagat	**TGTAGA**	gtgactggagttcagacgtgtgctcttccgatct	GGACTACHVGGGTWTCTAAT
V4R	42	caagcagaagacggcatacgagat	**ATCAGA**	gtgactggagttcagacgtgtgctcttccgatct	GGACTACHVGGGTWTCTAAT
V4R	43	caagcagaagacggcatacgagat	**ACATGA**	gtgactggagttcagacgtgtgctcttccgatct	GGACTACHVGGGTWTCTAAT
V4R	44	caagcagaagacggcatacgagat	**TAGACA**	gtgactggagttcagacgtgtgctcttccgatct	GGACTACHVGGGTWTCTAAT
V4R	45	caagcagaagacggcatacgagat	**GAGAAT**	gtgactggagttcagacgtgtgctcttccgatct	GGACTACHVGGGTWTCTAAT
V4R	47	caagcagaagacggcatacgagat	**AGGTAT**	gtgactggagttcagacgtgtgctcttccgatct	GGACTACHVGGGTWTCTAAT
V4R	48	caagcagaagacggcatacgagat	**TTGCAT**	gtgactggagttcagacgtgtgctcttccgatct	GGACTACHVGGGTWTCTAAT
V4R	49	caagcagaagacggcatacgagat	**TGGATT**	gtgactggagttcagacgtgtgctcttccgatct	GGACTACHVGGGTWTCTAAT
V4R	50	caagcagaagacggcatacgagat	**ACCATT**	gtgactggagttcagacgtgtgctcttccgatct	GGACTACHVGGGTWTCTAAT
V4R	51	caagcagaagacggcatacgagat	**CTAGTT**	gtgactggagttcagacgtgtgctcttccgatct	GGACTACHVGGGTWTCTAAT
V4R	53	caagcagaagacggcatacgagat	**TCTCTT**	gtgactggagttcagacgtgtgctcttccgatct	GGACTACHVGGGTWTCTAAT
V4R	54	caagcagaagacggcatacgagat	**GTAAGT**	gtgactggagttcagacgtgtgctcttccgatct	GGACTACHVGGGTWTCTAAT
V4R	55	caagcagaagacggcatacgagat	**CAATGT**	gtgactggagttcagacgtgtgctcttccgatct	GGACTACHVGGGTWTCTAAT
V4R	57	caagcagaagacggcatacgagat	**ATGACT**	gtgactggagttcagacgtgtgctcttccgatct	GGACTACHVGGGTWTCTAAT
V4R	58	caagcagaagacggcatacgagat	**ACTTCT**	gtgactggagttcagacgtgtgctcttccgatct	GGACTACHVGGGTWTCTAAT
V4R	59	caagcagaagacggcatacgagat	**CATAAG**	gtgactggagttcagacgtgtgctcttccgatct	GGACTACHVGGGTWTCTAAT

V4R	60	caagcagaagacggcatacgagat	**TTCTAG**	gtgactggagttcagacgtgtgctcttccgatct	GGACTACHVGGGTWTCTAAT
V4R	61	caagcagaagacggcatacgagat	**AAGATG**	gtgactggagttcagacgtgtgctcttccgatct	GGACTACHVGGGTWTCTAAT
V4R	62	caagcagaagacggcatacgagat	**TATGTG**	gtgactggagttcagacgtgtgctcttccgatct	GGACTACHVGGGTWTCTAAT
V4R	63	caagcagaagacggcatacgagat	**AATTGG**	gtgactggagttcagacgtgtgctcttccgatct	GGACTACHVGGGTWTCTAAT
V4R	64	caagcagaagacggcatacgagat	**TAATCG**	gtgactggagttcagacgtgtgctcttccgatct	GGACTACHVGGGTWTCTAAT
V4R	65	caagcagaagacggcatacgagat	**ACTAAC**	gtgactggagttcagacgtgtgctcttccgatct	GGACTACHVGGGTWTCTAAT
V4R	66	caagcagaagacggcatacgagat	**TGTTAC**	gtgactggagttcagacgtgtgctcttccgatct	GGACTACHVGGGTWTCTAAT
V4R	67	caagcagaagacggcatacgagat	**ATACAC**	gtgactggagttcagacgtgtgctcttccgatct	GGACTACHVGGGTWTCTAAT
V4R	68	caagcagaagacggcatacgagat	**CTTATC**	gtgactggagttcagacgtgtgctcttccgatct	GGACTACHVGGGTWTCTAAT
V4R	69	caagcagaagacggcatacgagat	**AGATTC**	gtgactggagttcagacgtgtgctcttccgatct	GGACTACHVGGGTWTCTAAT
V4R	71	caagcagaagacggcatacgagat	**TGCGAA**	gtgactggagttcagacgtgtgctcttccgatct	GGACTACHVGGGTWTCTAAT
V4R	73	caagcagaagacggcatacgagat	**CTGTCA**	gtgactggagttcagacgtgtgctcttccgatct	GGACTACHVGGGTWTCTAAT
V4R	74	caagcagaagacggcatacgagat	**GCAGAT**	gtgactggagttcagacgtgtgctcttccgatct	GGACTACHVGGGTWTCTAAT
V4R	75	caagcagaagacggcatacgagat	**TCGTGT**	gtgactggagttcagacgtgtgctcttccgatct	GGACTACHVGGGTWTCTAAT
V4R	76	caagcagaagacggcatacgagat	**GAACCT**	gtgactggagttcagacgtgtgctcttccgatct	GGACTACHVGGGTWTCTAAT
V4R	77	caagcagaagacggcatacgagat	**GTCATG**	gtgactggagttcagacgtgtgctcttccgatct	GGACTACHVGGGTWTCTAAT
V4R	78	caagcagaagacggcatacgagat	**GATAGC**	gtgactggagttcagacgtgtgctcttccgatct	GGACTACHVGGGTWTCTAAT
V4R	79	caagcagaagacggcatacgagat	**AAGTCC**	gtgactggagttcagacgtgtgctcttccgatct	GGACTACHVGGGTWTCTAAT
V4R	80	caagcagaagacggcatacgagat	**ATTGCC**	gtgactggagttcagacgtgtgctcttccgatct	GGACTACHVGGGTWTCTAAT
V4R	81	caagcagaagacggcatacgagat	**CCGAGA**	gtgactggagttcagacgtgtgctcttccgatct	GGACTACHVGGGTWTCTAAT
V4R	82	caagcagaagacggcatacgagat	**CGCTGA**	gtgactggagttcagacgtgtgctcttccgatct	GGACTACHVGGGTWTCTAAT
V4R	83	caagcagaagacggcatacgagat	**GGCACA**	gtgactggagttcagacgtgtgctcttccgatct	GGACTACHVGGGTWTCTAAT
V4R	84	caagcagaagacggcatacgagat	**CGTGCA**	gtgactggagttcagacgtgtgctcttccgatct	GGACTACHVGGGTWTCTAAT
V4R	85	caagcagaagacggcatacgagat	**GGCCTT**	gtgactggagttcagacgtgtgctcttccgatct	GGACTACHVGGGTWTCTAAT
V4R	86	caagcagaagacggcatacgagat	**CCTGGT**	gtgactggagttcagacgtgtgctcttccgatct	GGACTACHVGGGTWTCTAAT
V4R	87	caagcagaagacggcatacgagat	**CAGGCT**	gtgactggagttcagacgtgtgctcttccgatct	GGACTACHVGGGTWTCTAAT
V4R	88	caagcagaagacggcatacgagat	**GTCGCT**	gtgactggagttcagacgtgtgctcttccgatct	GGACTACHVGGGTWTCTAAT
V4R	89	caagcagaagacggcatacgagat	**GCGTAG**	gtgactggagttcagacgtgtgctcttccgatct	GGACTACHVGGGTWTCTAAT

V4R	90	caagcagaagacggcatacgagat	**CTGGAG**	gtgactggagttcagacgtgtgctcttccgatct	GGACTACHVGGGTWTCTAAT
V4R	91	caagcagaagacggcatacgagat	**CTACGG**	gtgactggagttcagacgtgtgctcttccgatct	GGACTACHVGGGTWTCTAAT
V4R	92	caagcagaagacggcatacgagat	**ACACCG**	gtgactggagttcagacgtgtgctcttccgatct	GGACTACHVGGGTWTCTAAT
V4R	93	caagcagaagacggcatacgagat	**GTTCCG**	gtgactggagttcagacgtgtgctcttccgatct	GGACTACHVGGGTWTCTAAT
V4R	94	caagcagaagacggcatacgagat	**CAGCAC**	gtgactggagttcagacgtgtgctcttccgatct	GGACTACHVGGGTWTCTAAT
V4R	95	caagcagaagacggcatacgagat	**CCGTTC**	gtgactggagttcagacgtgtgctcttccgatct	GGACTACHVGGGTWTCTAAT
V4R	96	caagcagaagacggcatacgagat	**GCATCC**	gtgactggagttcagacgtgtgctcttccgatct	GGACTACHVGGGTWTCTAAT
V4R	97	caagcagaagacggcatacgagat	**TACGCC**	gtgactggagttcagacgtgtgctcttccgatct	GGACTACHVGGGTWTCTAAT
